# Nano-Fe₃O₄ enhances salt–alkali tolerance of melon seedlings via inhibition of *CmBHLH93* and activation of ABA/JA signaling, antioxidant defense, and maintenance of Na^+^/K^+^ homeostasis

**DOI:** 10.1093/hr/uhag229

**Published:** 2026-06-05

**Authors:** Zhanming Tan, Lvjun Cui, Yuquan Peng, Qi Li, Zhilong Bie

**Affiliations:** National Key Laboratory for Germplasm Innovation & Utilization of Horticultural Crops/College of Horticulture and Forestry Sciences, Huazhong Agricultural University, Wuhan 430070, China; Xinjiang Production & Construction Corps Key Laboratory of Facility Agriculture/College of Horticulture and Forestry, Tarim University, Alar 843300, China; National Key Laboratory for Germplasm Innovation & Utilization of Horticultural Crops/College of Horticulture and Forestry Sciences, Huazhong Agricultural University, Wuhan 430070, China; Hubei Key Laboratory of Spices & Horticultural Plant Germplasm Innovation & Utilization/College of Horticulture and Gardening, Yangtze University, Jingzhou 434022, China; Xinjiang Production & Construction Corps Key Laboratory of Facility Agriculture/College of Horticulture and Forestry, Tarim University, Alar 843300, China; National Key Laboratory for Germplasm Innovation & Utilization of Horticultural Crops/College of Horticulture and Forestry Sciences, Huazhong Agricultural University, Wuhan 430070, China; Xinjiang Production & Construction Corps Key Laboratory of Facility Agriculture/College of Horticulture and Forestry, Tarim University, Alar 843300, China; Hubei Hongshan Laboratory, Wuhan 430070, China

## Abstract

Soil salinization and alkalinization threaten plant growth worldwide. In response, plants deploy various physiological and molecular defense mechanisms. Nanobiotechnology offers a potential strategy to enhance crop stress tolerance. Here, we found that nano-Fe₃O₄ increased salt–alkali tolerance in melon seedlings by suppressing the expression of the transcription factor *CmBHLH93*. Transcriptomic analysis revealed strong downregulation of *CmBHLH93* upon nano-Fe₃O₄ application under salt–alkali stress. Functional assays showed that CmBHLH93 acted as a negative regulator of salt tolerance. It directly bound the promoters of key defense-related genes involved in abscisic acid (ABA) synthesis (*CmNCED3*), jasmonic acid (JA) synthesis (*CmAOS*), antioxidant activity (*CmPOD-1*), and Na^+^ transport (*CmSOS1*), thereby repressing their transcription. Taken together, nano-Fe₃O₄ enhanced melon salt tolerance by downregulating *CmBHLH93* transcription and activating downstream responses associated with ABA/JA signaling, antioxidant capacity, and Na^+^/K^+^ homeostasis. These results define a regulatory mechanism through which nanomaterials modulate plant tolerance to salt–alkali stress and provide a conceptual basis for improving crop stress tolerance using nanotechnology.

## Introduction

Soil salinization and alkalinization have become an increasingly serious global problem driven by population growth and environmental degradation [1, 2]. The main harmful salts in soils include NaCl, Na₂SO₄, NaHCO₃, and Na₂CO₃. In China, more than half of affected soils contain both neutral and alkaline salts, making saline–alkali stress as a prevalent combined constraint [3]. Saline–alkali stress comprises two components: (i) salt stress, mainly caused by neutral salts such as NaCl and Na₂SO₄, and (ii) alkaline stress, driven by NaHCO₃ and Na₂CO₃ and characterized by elevated pH [4]. Salt stress primarily induces osmotic stress and ion toxicity [5, 6]. High salt concentrations reduce soil water potential, thereby hindering water uptake by plant roots [7]. Concurrently, excessive accumulation of Na^+^ and Cl^+^ in cells disrupts ionic homeostasis and inhibits multiple metabolic enzymes [8, 9]. In contrast, alkaline stress additionally imposes high-pH stress. Elevated soil pH limits the availability of essential mineral nutrients, including Fe^2+^, Mg^2+^, Ca^2+^, and PO₄^3−^, as these elements precipitate as hydroxides or carbonates that are poorly accessible to roots [10, 11]. High pH can also directly impair cell walls and plasma membranes, thereby disrupting the proton gradient required for cellular function [12]. Moreover, osmotic stress, ion toxicity, and high pH collectively promote the overproduction of reactive oxygen species (ROS), resulting in oxidative damage that restricts growth and development [2, 13]. Despite this complexity, most studies have focused primarily on salt stress, whereas salt–alkali stress has received comparatively limited attention.

To cope with saline–alkali stress, plants have evolved a complex array of physiological and molecular mechanisms [14]. In parallel, plant nanobiotechnology has shown great potential in enhancing crop stress tolerance [15]. Due to their high surface-area-to-volume ratio and unique physicochemical properties, many nanomaterials have been explored to improve plant salt tolerance [16, 17]. Among these, iron (Fe) is an essential micronutrient that has been reported to enhance salt tolerance, partly through the regulation of antioxidant systems [18]. Nano-Fe serves as an efficient iron source, alleviating iron deficiency in plants under saline–alkali stress [19], restoring chlorophyll synthesis, and improving photosynthetic efficiency in muskmelon plants [20]. In addition, iron functions as a cofactor for key antioxidant enzymes, including peroxidase (POD) and catalase (CAT), and is thus critical for their activity [21]. In *Moringa*, foliar application of 60 mg/kg ZnO and Fe₃O₄ nanoparticles (NPs) increases chlorophyll content and antioxidant enzyme activity, thereby mitigating salt stress effects [22]. In sunflower, foliar application of FeSO_4_ NPs enhances polyphenol oxidase, CAT, and POD activities, reducing ROS accumulation [23]. Fe-based NPs have also been shown to help maintain a lower Na^+^/K^+^ ratio. For instance, Fe NP treatment reduces Na^+^ accumulation in the shoots of wheat and grapevine under salt stress while increasing K^+^ uptake [24, 25]. In *Trachyspermum ammi*, combined foliar application of salicylic acid (SA) and nano-Fe₂O₃ increases K^+^ and Fe content, elevates endogenous SA levels, and enhances antioxidant enzyme activity, thereby enhancing salt tolerance [26]. These results suggest that nano-Fe-mediated stress tolerance may involve modulation of phytohormone pathways. Despite these promising effects, the safety of nanomaterials remains a critical concern for their agricultural application. Generally, Fe₃O₄ NPs exhibit relatively good biocompatibility and lower toxicity compared to other metallic nanoparticles [27, 28]. However, their effects on plants are highly dose-dependent. Excessive accumulation of iron-based nanoparticles may trigger Fenton reactions, leading to overproduction of ROS and consequent oxidative damage [29, 30]. Additionally, high NP concentrations may physically obstruct root tissues or interfere with nutrient uptake pathways [31, 32]. Therefore, evaluating potential phytotoxicity and defining safe concentration ranges are essential prerequisites for the application of Fe₃O₄ NPs. Although Fe-based NPs show clear potential for mitigating salinity stress, the underlying molecular mechanisms remain poorly understood.

Transcription factors act as central regulators in plants, controlling gene expression and mediating responses to environmental stresses [33]. Basic helix–loop–helix proteins (bHLHs) constitute one of the largest transcription factor families and play central roles in plant growth, development, and stress responses [34]. bHLH transcription factors are particularly important in iron uptake and iron homeostasis [35]. Under iron-deficient conditions caused by high pH, plants activate a set of bHLH transcription factors, including *FIT*, *bHLH38*, and *bHLH39* in *Arabidopsis* [36]. Some bHLH proteins, such as IRO2, IRO3, and FIT, form heterodimers or homodimers to regulate the expression of genes involved in iron uptake, thereby enhancing iron acquisition and transport in roots [37, 38]. More broadly, bHLH transcription factors are central regulators of other abiotic stress responses, including salt stress [39]. They contribute to salt tolerance by modulating antioxidant systems, ion homeostasis, and hormone-signaling pathways [40]. Specifically, bHLHs are extensively involved in ABA and jasmonic acid (JA) signaling, which are crucial for stress adaptation. For instance, MYC2, a well-characterized bHLH transcription factor, functions as a master regulator of JA signaling and controls the expression of stress-responsive genes [41, 42]. Similarly, several bHLH transcription factors regulate ABA biosynthesis or signaling, thereby controlling stomatal closure and downstream stress responses. In maize, ZmbHLH32 modulates salt tolerance through the ZmIAA9-ZmARF1 module [43]. In *Moso bamboo*, a set of *PebHLH* genes containing ABA- and JA-responsive *cis*-elements is upregulated in response to stress and hormonal signals [44].

bHLH transcription factors enhance salt tolerance primarily by increasing the activity of antioxidant enzyme activity and reducing ROS accumulation under salt stress in *Arabidopsis* [45], maize [46], and wheat [47]. Maintaining Na^+^/K^+^ homeostasis is crucial for plant survival under saline conditions. This process largely relies on the Salt Overly Sensitive (SOS) pathway, particularly the plasma membrane Na^+^/H^+^ antiporter SOS1 [48, 49]. SOS1 exports excess Na^+^ from the cytoplasm into the apoplast using the proton gradient, thereby preventing Na^+^ toxicity and safeguarding K^+^ uptake [50]. Notably, some bHLH factors can directly regulate ion transporters. In rice, OsbHLH044 positively regulates salt stress responses by activating ion homeostasis–related genes, including *OsHKT1;3*, *OsHAK7*, *OsSOS1*, and *OsNHX2*, thereby reducing Na^+^ accumulation and maintaining K^+^ balance [51]. However, most studies to date have focused on bHLH functions under either salt stress or iron deficiency. It remains largely unclear whether, and how, bHLH transcription factors participate in regulatory networks mediated by exogenous nanomaterials such as nano-Fe₃O₄ under combined salt–alkali stress. In particular, the molecular mechanisms linking bHLH-mediated iron homeostasis to salt–alkali tolerance in the context of nanomaterial exposure remain to be elucidated.

In our previous study, Fe₃O₄ nanomaterials were identified as a strategy to enhance melon tolerance under salt–alkali stress, and 100 mg/L was established as the optimal application concentration. At this level, nano-Fe₃O₄ improved salt–alkali tolerance in melon seedlings by regulating hormone balance, optimizing ion homeostasis, antioxidant capacity, and organic acid metabolism [52]. Given the known roles of bHLHs in linking iron homeostasis and stress responses, we hypothesized a functional connection between bHLH-mediated regulation and nano-Fe₃O₄–induced stress adaptation. In this study, exogenous nano Fe₃O₄ significantly alleviated salt–alkali injury in melon seedlings. Transcriptomic analysis identified the bHLH transcription factor *CmBHLH93* whose expression was markedly reduced following nano-Fe₃O₄ treatment. Functional validation confirmed that CmBHLH93 acted as a negative regulator of salt tolerance. Further analyses revealed that CmBHLH93 directly bound to the promoters of key defense genes involved in ABA synthesis, JA synthesis, antioxidant defense, and ion transport, thereby repressing their transcription. Collectively, these results indicate that nano Fe₃O₄ enhances melon salt tolerance by suppressing *CmBHLH93* expression, thereby activating downstream defense responses.

## Results

### Screening of key genes for nano-Fe_3_O_4_–enhanced salt–alkali tolerance in melon seedlings

Previous studies indicate that roots are the primary site of response to salt–alkali stress in melon seedlings. To identify root genes associated with nano-Fe_3_O_4_–mediated tolerance, differentially expressed genes (DEGs) were analyzed. Under salt–alkali stress, 1492 DEGs were identified in roots of nano-Fe₃O₄–treated seedlings compared with water-treated controls. Gene Ontology (GO) enrichment analysis of these DEGs (Fig. 1a) identified 32 transcription factors. Expression profiling (Fig. 1b) showed that only two transcription factors, *CmBHLH93* and *CmGATA4*, were downregulated. After nano-Fe_3_O_4_ treatment, their expression decreased by 70.3% and 53.8%, respectively (Fig. 1c and d), suggesting that nano-Fe_3_O_4_ may enhance salt–alkali tolerance by repressing these transcription factors. Given the stronger downregulation of *CmBHLH93*, it was prioritized for further investigation. Further proteomic analysis (Table S1) revealed that, following 5 h of treatment with 200 mM salt–alkali solution, the relative protein abundance of CmBHLH93 in the roots of melon seedlings upon nano-Fe₃O₄ treatment was significantly reduced by 51.8% compared to the water-treated controls (Fig. 1e).

**Figure 1:**
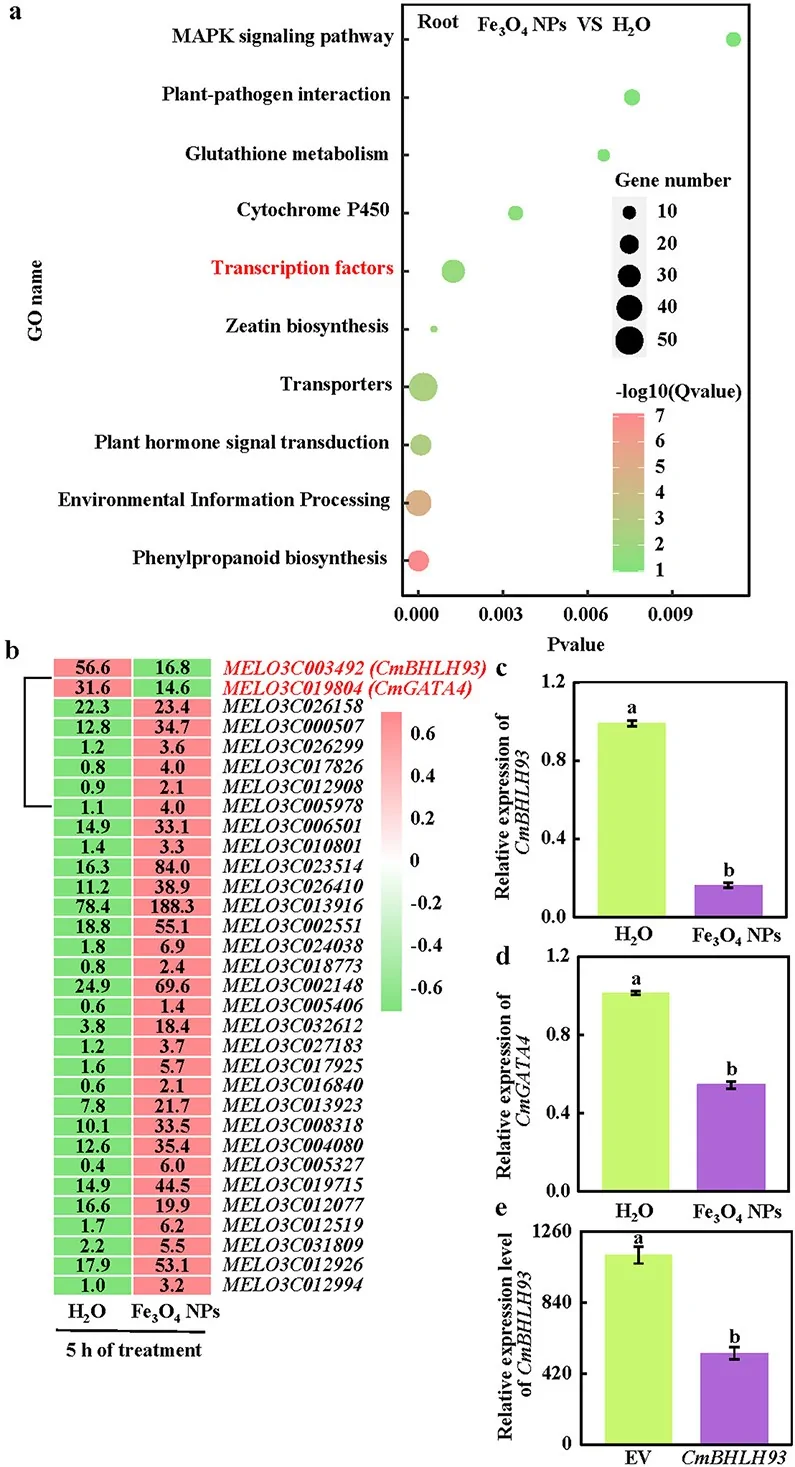
Screening and analysis of transcription factors in melon roots treated with nano-Fe_3_O_4_ under 5-h salt–alkali stress. (a) Gene Ontology (GO) enrichment analysis of differentially expressed genes (DEGs) in roots of melon seedlings exposed to 5-h salt–alkali stress (200 mM, molar ratio of NaCl:Na₂SO₄:NaHCO₃:Na₂CO₃ = 1:9:9:1, pH 8.9), following foliar application of 100 mg/L nano-Fe₃O₄ (Fe₃O₄ NPs) or distilled water (H_2_O) as a control. (b) Heatmap illustrating the expression patterns of the 32 transcription-factor DEGs in nano-Fe₃O₄-treated and control samples (*n* = 3 biological replicates per treatment). (c and d) Quantitative real-time PCR (qRT-PCR) validation of relative expression levels of *CmBHLH93* (c) and *CmGATA4* (d) in roots. (e) Relative CmBHLH93 protein level determined by LC-MS/MS. Data are presented as mean ± standard error of the mean (SEM; *n* = 3 biological replicates, each comprising six seedlings). Different lowercase letters denote statistically significant differences between treatments (*P* < 0.05) determined by Duncan’s new multiple range test (DMRT) following two-way ANOVA.

### Effect of *CmBHLH93* on nano-Fe_3_O_4_–enhanced salt–alkali tolerance in melon seedlings

Root-specific editing of *CmBHLH93* in transgenic melon seedlings was assessed to evaluate the efficiency of the CRISPR-Cas9 gene-editing system. Targeted mutations in *CmBHLH93* were confirmed by sequencing analysis in knockout lines (KOBHLH93), demonstrating effective genome editing (Fig. 2a). Root-specific expression of *CmBHLH93* was analyzed in overexpression lines (OEBHLH93), in which transcript levels were markedly higher than in empty vector (EV) controls (Fig. 2b). These results confirm successful generation of *CmBHLH93* knockout and overexpression lines for subsequent functional analysis.

**Figure 2:**
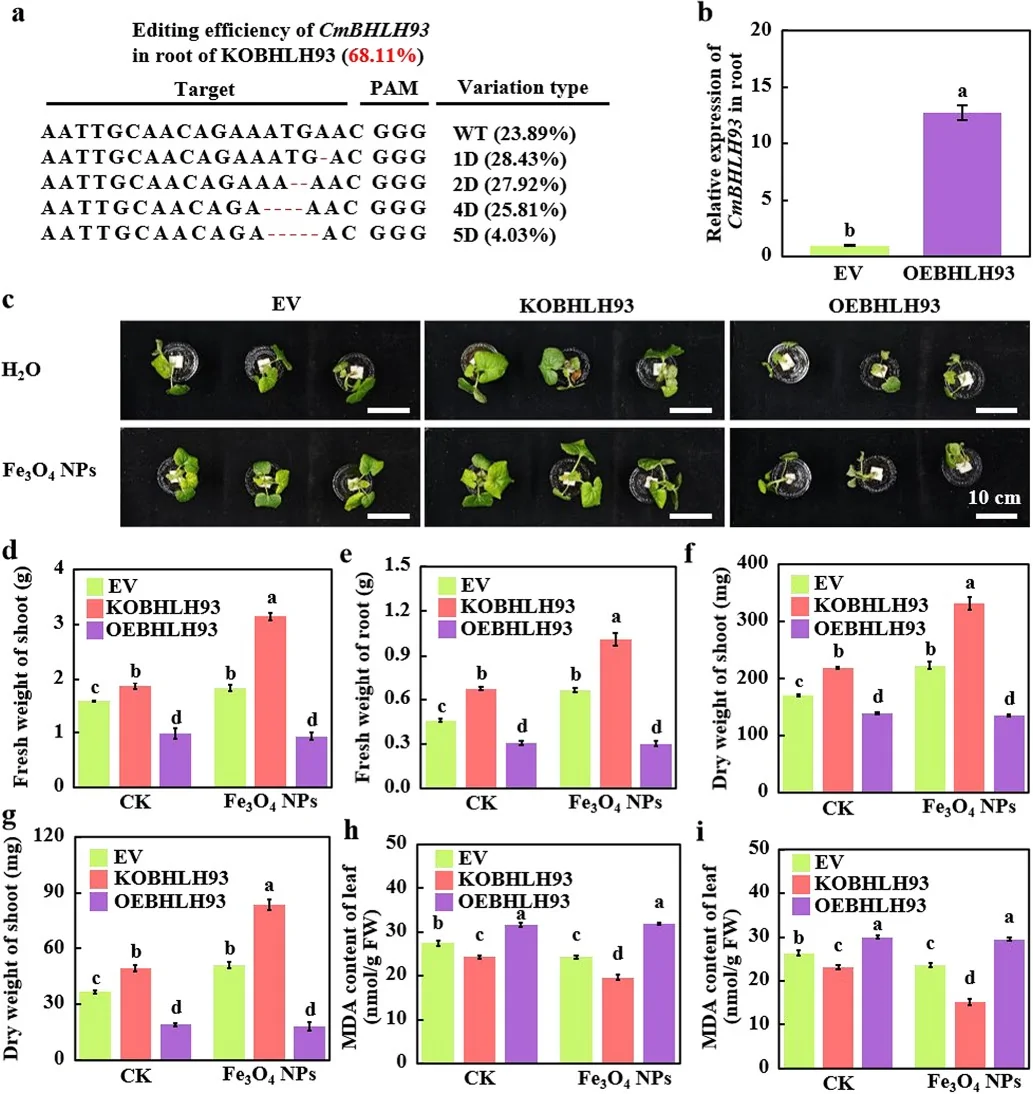
Effects of *CmBHLH93* knockout and overexpression on nano-Fe_3_O_4_–mediated regulation of melon seedling growth under salt–alkali stress. (a)Hi-TOM (high-throughput tracking of mutations) analysis confirming targeted insertion–deletion (indel) mutations at the *CmBHLH93* locus in CRISPR/Cas9-mediated knockout (KOBHLH93) root-transformed lines. (b) Relative transcript abundance of *CmBHLH93* in overexpression (OEBHLH93) root-transformed lines and empty vector (EV) controls, determined by qRT-PCR with *CmActin* as the internal reference. (c) Phenotypes of KOBHLH93, OEBHLH93, and EV seedlings after 5 days of salt–alkali stress (200 mM) with or without foliar nano-Fe₃O₄ application (100 mg/L, applied 24 h prior to stress imposition). Scale bar = 10 cm. (d–g) Quantification of shoot fresh weight (d), root fresh weight (e), shoot dry weight (f), and root dry weight (g) after oven-drying at 80°C to constant weight. (h and i) Malondialdehyde (MDA) content in leaves (h) and roots (i). For (b–i), data are presented as mean ± standard error of the mean (SEM; *n* = 3 biological replicates, each comprising six seedlings). Statistical significance was determined by two-way ANOVA followed by Duncan’s new multiple range test (DMRT); different lowercase letters indicate significant differences between treatments (*P* < 0.05).

To investigate the effects of root-specific knockout and overexpression of *CmBHLH93* on nano-Fe₃O₄–mediated regulation of salt–alkali tolerance, plant growth parameters were measured. Under 200 mM salt–alkali stress with water treatment, *CmBHLH93* knockout significantly promoted seedling growth compared to EV controls (Fig. 2c). Shoot fresh weight (Fig. 2d), root fresh weight (Fig. 2e), shoot dry weight, and root dry weight (Fig. 2f and g) increased by 17.0%, 46.7%, 29.2%, and 34.3%, respectively. In contrast, *CmBHLH93* overexpression significantly inhibited growth, with corresponding decreases of 37.8%, 33.0%, 18.3%, and 47.4%, indicating a negative association of *CmBHLH93* expression with salt–alkali tolerance in melon. Under the same stress conditions, nano-Fe₃O₄ treatment further promoted growth in the knockout lines compared with EV controls, with increases in shoot fresh weight (Fig. 2d), root fresh weight (Fig. 2e), shoot dry weight, and root dry weight (Fig. 2f and g) of 72.7%, 52.0%, 48.1%, and 62.4%, respectively. Conversely, overexpression lines showed significant growth inhibition, with corresponding reductions of 48.8%, 54.5%, 40.0%, and 64.3%. Additionally, under salt–alkali stress with nano-Fe₃O₄ application, malondialdehyde (MDA) content in leaves and roots of knockout seedlings (Fig. 2h and i) was lower than in EV controls. Together, these results indicate that nano-Fe₃O₄ enhances salt–alkali tolerance in melon, at least in part, by suppressing *CmBHLH93* expression, consistent with its role as a negative regulator.

### Effect of *CmBHLH93* on gene expression in melon seedlings treated with nano-Fe₃O₄ under salt–alkali stress

Previous studies showed that foliar application of nano-Fe₃O₄ effectively alleviates wilting in melon seedlings after 5 h of salt–alkali stress. To characterize early molecular responses, transcriptome sequencing was performed on leaves and roots of melon seedlings exposed to 5 h salt–alkali stress with nano-Fe₃O₄ application. Principal component analysis (PCA) showed tight clustering of biological replicates within the 95% confidence intervals (Fig. 3a and b), supporting data reproducibility. Differential expression analysis showed that, in leaves (Fig. 3c), knockout seedlings exhibited 1045 upregulated and 1117 downregulated genes, whereas overexpression seedlings displayed 1182 upregulated and 1333 downregulated genes relative to root-transformed EV controls. In roots (Fig. 3d), knockout seedlings showed 361 upregulated and 421 downregulated genes, whereas overexpression seedlings showed 105 upregulated and 356 downregulated genes.

**Figure 3:**
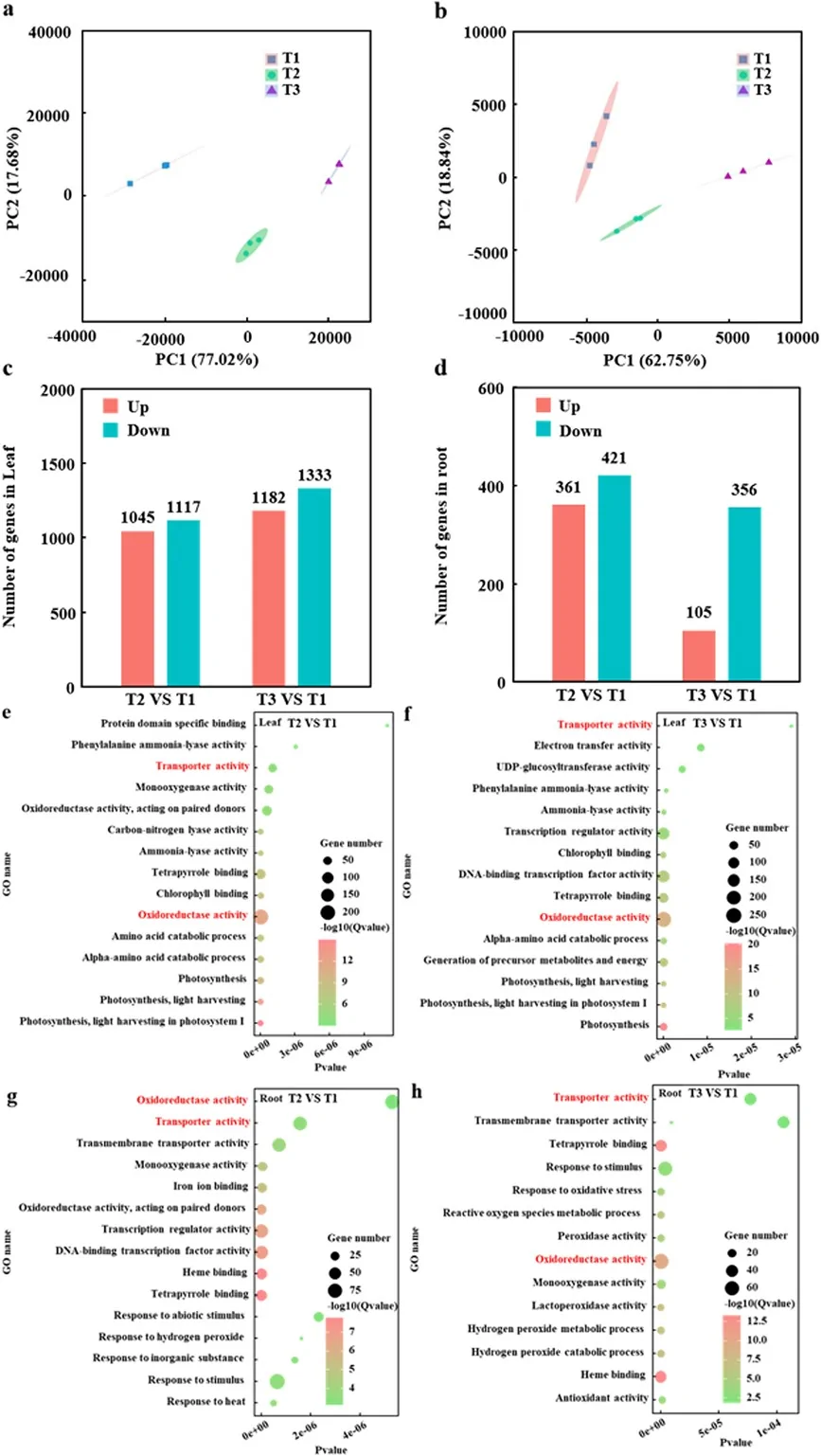
Effect of *CmBHLH93* on gene expression in melon seedlings treated with nano-Fe₃O₄ under 5-h salt–alkali stress. (a and b) Principal component analysis (PCA) of RNA sequencing data from shoots (a) and roots (b) of KOBHLH93, OEBHLH93, and EV seedlings. Ellipses represent 95% confidence intervals for each genotype cluster based on the first two principal components (PC1 and PC2). (c and d) Volcano plots depicting the distribution of DEGs in shoots (c) and roots (d). DEGs were defined as genes with |log₂(fold change)| ≥ 1 and adjusted *P* < 0.05. (e–h) GO enrichment analysis of DEGs categorized by molecular function in KOBHLH93 versus EV (e, shoots; g, roots) and OEBHLH93 versus EV (f, shoots; h, roots). Treatment designations: T1, EV + nano-Fe₃O₄ + salt–alkali; T2, KOBHLH93 + nano-Fe₃O₄ + salt–alkali; T3, OEBHLH93 + nano-Fe₃O₄ + salt–alkali.

To explore the regulatory role of *CmBHLH93* in salt–alkali tolerance, GO (Gene Ontology) and KEGG (Kyoto Encyclopedia of Genes and Genomes) enrichment analyses were performed. Both knockout and overexpression seedlings showed GO enrichment in transporter activity and redox-related functions in leaves and roots (Fig. 3e–h). KEGG analysis further indicated significant enrichment in plant hormone signal transduction (Fig. S1). Together, these results suggest that *CmBHLH93* acts as a regulator in nano-Fe₃O₄–mediated responses to salt–alkali stress, potentially through modulation of redox processes, ion transport, and hormone signaling.

### Effect of *CmBHLH93* on abscisic acid and jasmonic contents and related gene expression in melon seedlings treated with nano-Fe₃O₄ under salt–alkali stress

To investigate the role of *CmBHLH93* in hormone metabolism under nano-Fe₃O₄–mediated salt–alkali stress, ABA and JA contents were measured. Under 200 mM salt–alkali stress with water treatment, *CmBHLH93* knockout significantly increased ABA and JA levels in leaves and roots compared with EV controls, with increases of 16.8%, 5.5%, 17.5%, and 25.0% for leaf ABA, root ABA, leaf JA, and root JA, respectively (Fig. 4a–d). Under salt–alkali stress with nano-Fe₃O₄ treatment, *CmBHLH93* overexpression significantly reduced hormone accumulation, with decreases of 19.7%, 25.3%, 25.4%, and 56.3%, respectively. In contrast, *CmBHLH93* knockout further increased in ABA and JA levels relative to EV controls, whereas overexpression lines showed the opposite trend. These results indicate that nano-Fe₃O₄ promotes ABA and JA accumulation by suppressing *CmBHLH93* expression.

**Figure 4:**
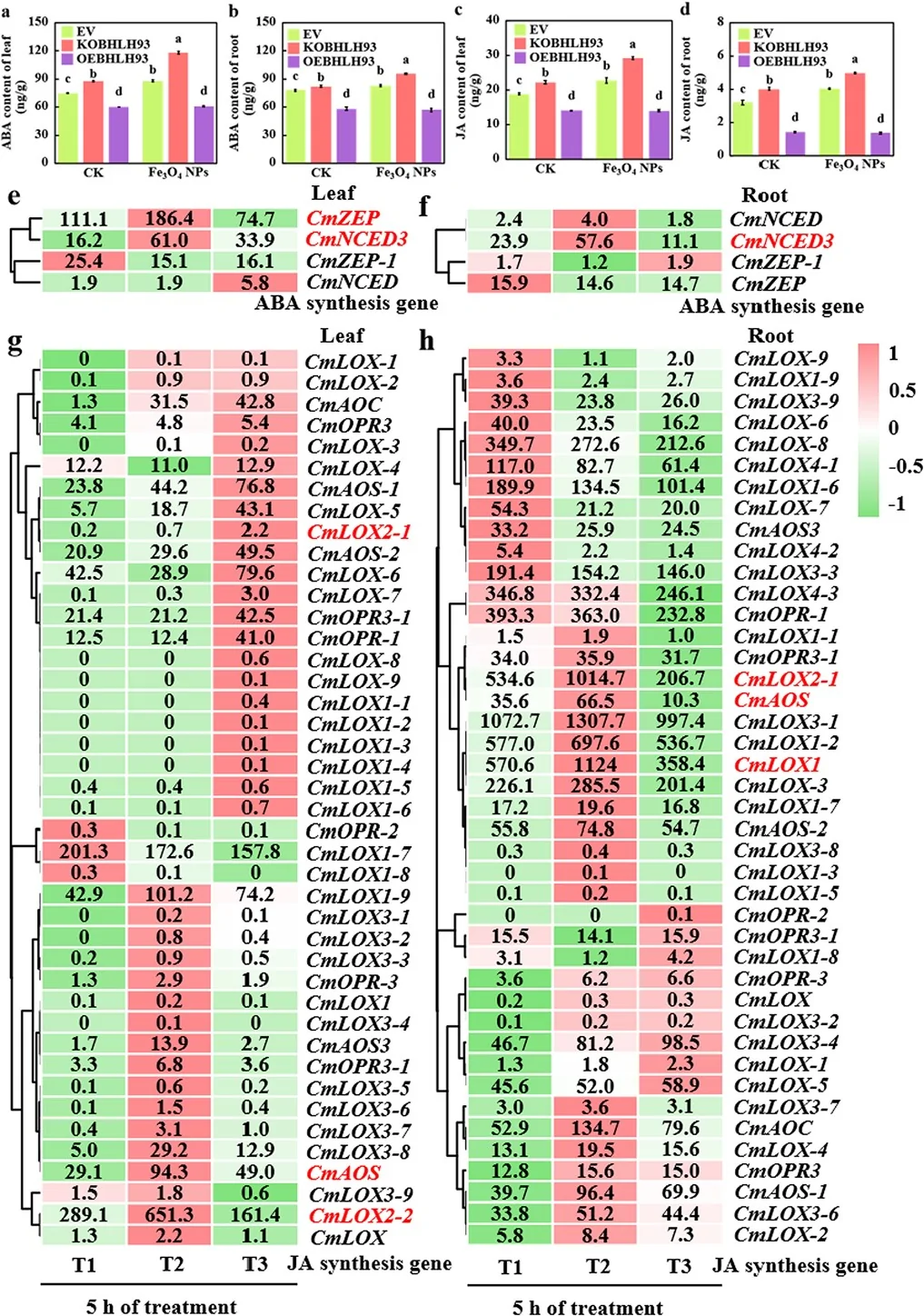
CmBHLH93 regulates ABA and JA contents and related gene expression in melon seedlings treated with nano-Fe₃O₄ under 5-h salt–alkali stress. (a–d) Endogenous hormone concentrations quantified by ultra-high-performance liquid chromatography–tandem mass spectrometry (UHPLC-MS/MS) with isotope-labeled internal standards: ABA in shoots (a) and roots (b); JA in shoots (c) and roots (d). (e and f) Expression levels of ABA biosynthetic genes in shoots (e) and roots (f): *CmZEP* (zeaxanthin epoxidase, MELO3C020872) and *CmNCED3* (9-*cis*-epoxycarotenoid dioxygenase, MELO3C027057). (g and h) Expression levels of JA biosynthetic genes in shoots (g) and roots (h): *CmLOX1* (lipoxygenase, MELO3C004245), *CmAOS* (allene oxide synthase, MELO3C018413), *CmAOC* (allene oxide cyclase), and *CmOPR* (12-oxophytodienoate reductase). Gene expression was determined by qRT-PCR with *CmActin* as the reference gene. All data are presented as mean ± standard error of the mean (SEM; *n* = 3 biological replicates). Statistical significance was determined by two-way ANOVA followed by Duncan’s new multiple range test (DMRT); different lowercase letters indicate significant differences between treatments (*P* < 0.05). Salt–alkali stress was applied at 200 mM with a molar ratio of NaCl:Na₂SO₄:NaHCO₃:Na₂CO₃ = 1:9:9:1.

To further identify downstream targets, expression of ABA and JA biosynthesis genes were further analyzed. In leaves (Fig. 4e–g), compared with EV controls, *CmBHLH93* knockout increased the expression of *MELO3C020872* (*CmZEP*) and *MELO3C027057* (*CmNCED3*) (Fig. S2a), whereas overexpression reduced their expression. In roots (Fig. 4f–h), *CmBHLH93* knockout upregulated *MELO3C027057* (*CmNCED3*)*, MELO3C004245* (*CmLOX1*), and *MELO3C018413* (*CmAOS*) (Fig. S2a–c), while overexpression downregulated these genes. Together, these results demonstrate that CmBHLH93 regulates the expression of key ABA and JA biosynthesis genes in both leaves and roots, thereby modulating ABA and JA accumulation and contributing to nano-Fe₃O₄–enhanced salt–alkali tolerance in melon seedlings.

### Effect of *CmBHLH93* on antioxidant enzyme activities and related gene expression in melon seedlings treated with nano-Fe₃O₄ under salt–alkali stress

To evaluate the role of *CmBHLH93* in oxidative stress responses under nano-Fe₃O₄–mediated salt–alkali stress, activities of multiple antioxidant enzymes were measured. Under 200 mM salt–alkali stress with water treatment, compared with EV controls, *CmBHLH93* knockout significantly increased leaf SOD, root POD, and root CAT activities (Fig. 5a, d, f) by 13.2%, 13.4%, and 7.7%, respectively, whereas other enzyme activities showed no significant changes (Fig. 5b, c, and  e). In contrast, *CmBHLH93* overexpression reduced leaf SOD, root POD, and root CAT activities by 12.1%, 22.9%, and 24.3%, respectively, supporting its negative regulatory role in salt–alkali tolerance. Under salt–alkali stress with nano-Fe₃O₄ treatment, *CmBHLH93* knockout further increased leaf SOD activity by 10.3% and root POD and CAT activities (Fig. 5d and f) by 8.6% and 7.5%, respectively, while other enzyme activities remained unchanged (Fig. 5c, e, and  b). Conversely, overexpression lines showed decreased leaf SOD activity (23.0%) and reduced root POD and CAT activities (31.6% and 31.0%, respectively).

**Figure 5:**
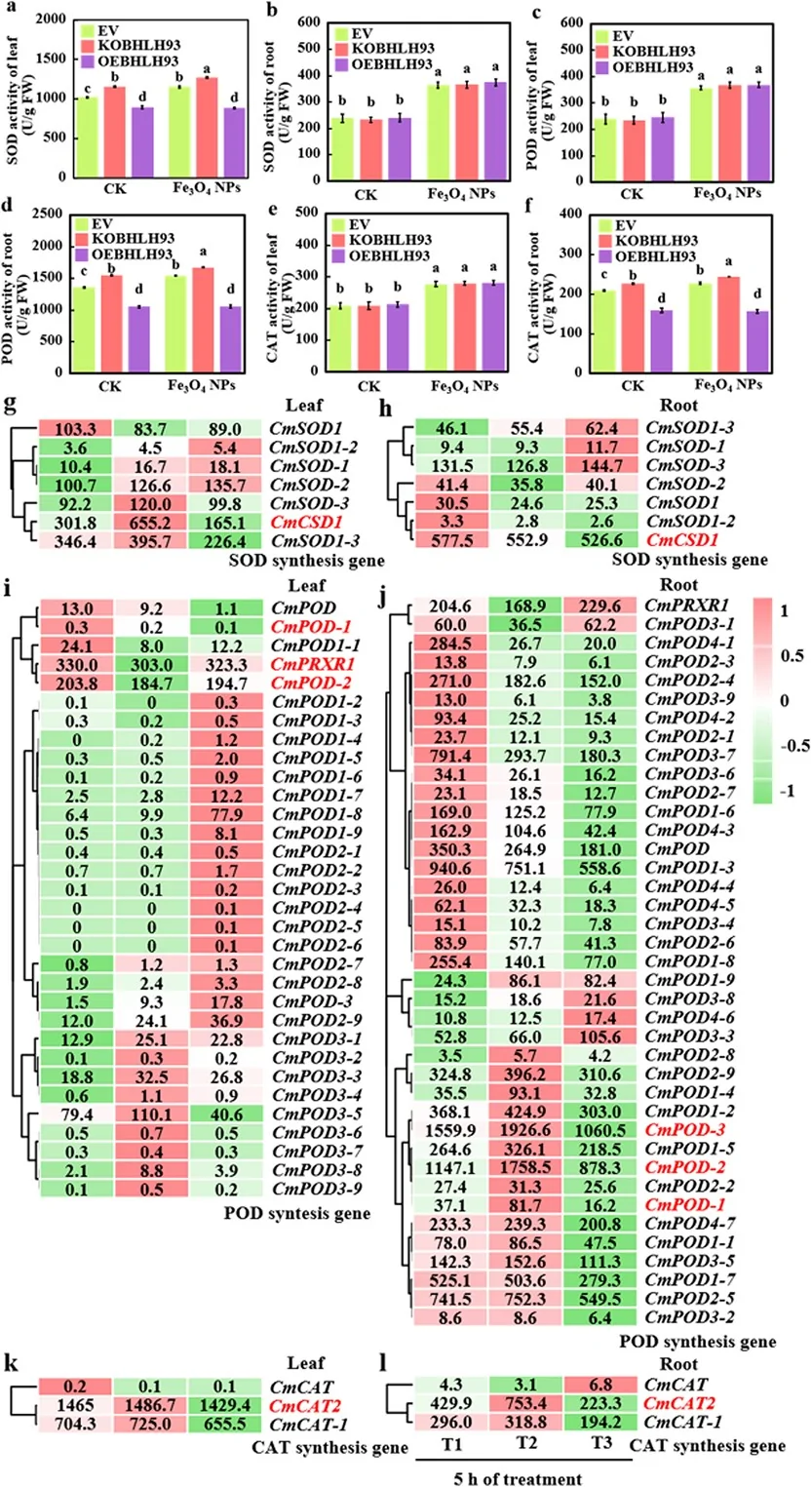
Effect of *CmBHLH93* on antioxidant enzyme activities and related gene expression in melon seedlings treated with nano-Fe₃O₄ under 5-h salt–alkali stress. (a and b) Superoxide dismutase (SOD) activity in shoots (a) and roots (b). (c and d) Peroxidase (POD) activity in shoots (c) and roots (d). (e and f) Catalase (CAT) activity in shoots (e) and roots (f). (g and h) Expression of SOD-encoding genes in shoots (g) and roots (h): *CmCSD1* (Cu/Zn-SOD, MELO3C015374) and *CmSOD* (Mn-SOD). (i and j) Expression of POD-encoding genes in shoots (i) and roots (j): *CmPOD-1* (MELO3C014650) and *CmPOD-2* (MELO3C009329). (k and l) Expression of CAT-encoding genes in shoots (k) and roots (l): *CmCAT1* and *CmCAT2*. Data represent mean ± standard error of the mean (SEM; *n* = 3 biological replicates). Statistical significance was determined by two-way ANOVA followed by Duncan’s new multiple range test (DMRT); different lowercase letters indicate significant differences between treatments (*P* < 0.05). Treatment designations: T1, EV + nano-Fe₃O₄ + salt–alkali; T2, KOBHLH93 + nano-Fe₃O₄ + salt–alkali; T3, OEBHLH93 + nano-Fe₃O₄ + salt–alkali.

To examine transcriptional regulation, expression of antioxidant enzyme–related genes was analyzed. Under control conditions, antioxidant enzyme–related gene expression in *CmBHLH93* overexpression seedlings was significantly lower than that in *CmBHLH93* knockout seedlings (Fig. 5g–j). Under salt treatment, SOD genes were significantly upregulated in *CmBHLH93* overexpression seedlings, particularly in roots, including *MELO3C015374* (*CmCSD1*) (Fig. S2d). In contrast, POD genes were significantly upregulated in *CmBHLH93* knockout seedlings, especially in roots, including *MELO3C009329* (*CmPOD-2*) and *MELO3C014650* (*CmPOD-1*) (Fig. S2e and f). Under salt stress, SOD, POD, and CAT expression was significantly reduced in EV controls, while overexpression of *CmBHLH93* caused a substantial upregulation of these genes. Overall, these results suggest that CmBHLH93 negatively regulates antioxidant defence at both enzymatic activity and transcriptional levels, while nano-Fe₃O₄ enhances antioxidant capacity and salt–alkali tolerance through suppression of *CmBHLH93*.

### Effect of *CmBHLH93* on Na^+^ and K^+^ content in melon seedlings treated with nano-Fe₃O₄ under salt stress

To further clarify the role of CmBHLH93 in melon salt tolerance, Na^+^ and K^+^ contents and their ratios were analyzed. Under control conditions, compared with EV controls, *CmBHLH93* knockout (KOBHLH93) seedlings showed significantly decreased Na^+^ content in shoots and roots (Fig. 6a and b) and significantly increased K^+^ content (Fig. 6c and d), with shoot and root Na^+^/K^+^ ratios reduced by ~40% and 43%, respectively (Fig. 6e and f). In contrast, *CmBHLH93* overexpression (OEBHLH93) seedlings exhibited significantly increased Na^+^ content in shoots and roots (Fig. 6a and b) and decreased K^+^ content (Fig. 6c and d), resulting in elevated shoot and root Na^+^/K^+^ ratios by ~1.4-fold and 0.4-fold, respectively. Under salt stress with nano-Fe₃O₄ treatment, EV seedlings exhibited improved ion homeostasis with reduced Na^+^/K^+^ ratios in both shoots and roots. *CmBHLH93* knockout further strengthened this effect by reducing Na^+^ accumulation, increasing K^+^ content, and decreasing Na^+^/K^+^ ratios more strongly. In contrast, *CmBHLH93* overexpression had only a weak effect on Na^+^ and K^+^ levels, with a slight increase in Na^+^/K^+^ ratio in roots and negligible changes in shoots (Fig. 6a–f). Collectively, these results indicate that CmBHLH93 functions as a negative regulator of ionic homeostasis under salt stress. Its knockout improves Na^+^ exclusion and K^+^ retention, thereby maintaining a lower Na^+^/K^+^ ratio, whereas overexpression disrupts ion balance and reduces salt tolerance. Notably, nano-Fe₃O₄ application further enhanced the ability of *CmBHLH93* knockout seedlings to maintain Na^+^/K^+^ homeostasis, highlighting its role in modulating ion transport under stress.

**Figure 6:**
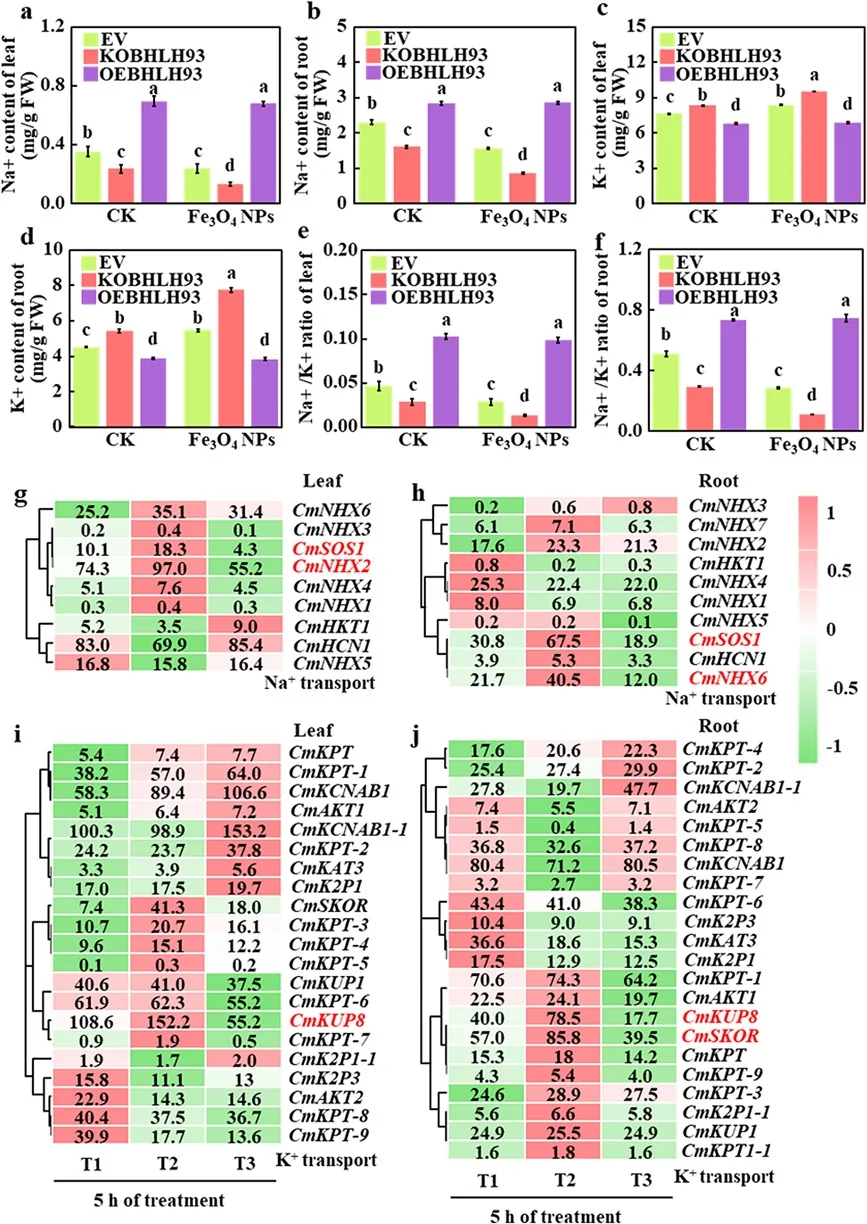
Effects of *CmBHLH93* on Na^+^ and K^+^ content in melon seedlings under 5-h salt stress after foliar application of nano-Fe₃O₄. (a and b) Sodium ion (Na^+^) content in shoots (a) and roots (b). (c and d) Potassium ion (K^+^) content in shoots (c) and roots (d). (e and f) Na^+^/K^+^ ratio in shoots (e) and roots (f). (g and h) Expression of Na^+^ transport-related genes in shoots (g) and roots (h): *CmSOS1* (plasma membrane Na^+^/H^+^ antiporter, MELO3C005228), *CmNHX* (vacuolar Na^+^/H^+^ exchanger), and *CmHKT1* (high-affinity K^+^ transporter 1). (i and j) Expression of K^+^ transport–related genes in shoots (i) and roots (j): *CmKUP8* (K^+^ uptake permease 8, MELO3C009941), *CmSKOR* (stellar K^+^ outward rectifier, MELO3C022820), *CmAKT1* (K^+^ channel AKT1), and *CmHCN1* (hyperpolarization-activated cyclic nucleotide-gated channel 1). Data are mean ± standard error of the mean (SEM; *n* = 3 biological replicates). Statistical significance was determined by two-way ANOVA followed by Duncan’s new multiple range test (DMRT); different lowercase letters indicate significant differences between treatments (*P* < 0.05). Treatment designations: T1, EV + nano-Fe₃O₄ + salt–alkali; T2, KOBHLH93 + nano-Fe₃O₄ + salt–alkali; T3, OEBHLH93 + nano-Fe₃O₄ + salt–alkali.

To investigate transcriptional regulation of ion transport, expression of Na^+^ and K^+^ transport–related genes were analyzed in seedlings treated with foliar-applied nano-Fe₃O₄ for 5 h. Under control conditions, compared with *CmBHLH93* knockout (KOBHLH93) seedlings, *CmBHLH93* overexpression (OEBHLH93) seedlings exhibited significant changes in the expression patterns of Na^+^ and K^+^-related genes in both shoots and roots (Fig. 6g–j). Under salt treatment, OEBHLH93 seedlings showed a significant upregulation of the Na^+^ transport–related gene (*MELO3C005228*, *CmSOS1*) (Fig. S2g), indicating enhanced Na^+^ transport and compartmentalization capacity. Additionally, the upregulation trend observed in K^+^ transport–related genes *(MELO3C009941, CmKUP8; MELO3C022820, CmSKOR)* (Fig. S2h and i) implies that *CmBHLH93* overexpression contributes to enhanced K^+^ uptake and translocation. Compared with the salt-treated control, the expressions of these genes in OEBHLH93 seedlings under salt stress were significantly higher. Overall, these data indicate that *CmBHLH93* modulates salt–alkali tolerance by regulating Na^+^ and K^+^ transport–related genes, thereby affecting ionic homeostasis under stress conditions.

### Interaction analysis of CmBHLH93 with downstream gene promoters

Subcellular localization analysis showed the nuclear localization of CmBHLH93 (Fig. 7a). Yeast transcriptional activation assays indicated that CmBHLH93 possessed intrinsic transcriptional activation activity (Fig. S3). Promoter analysis of key downstream genes identified putative CmBHLH93 binding sites (E-box) in the promoters of *CmNCED3*, *CmAOS*, *CmPOD-1*, and *CmSOS1*. The E-box-containing promoter fragments were cloned into LacZ reporter constructs and co-transformed with AD-*CmBHLH93* into yeast. Yeast harbouring both constructs formed blue colonies on SD/-Trp/-Ura/X-Gal medium, whereas the control yeast containing only the reporter plasmid showed no color development, indicating that CmBHLH93 can specifically interact with these promoter fragments (Fig. 7b). To further assess its transcriptional activity *in planta*, promoter–LUC (Luciferase) reporter constructs were co-expressed with *CmBHLH93* in *Nicotiana benthamiana* leaves. LUC fluorescence was significantly reduced in the presence of CmBHLH93 compared with promoter-LUC construct alone (Fig. 7c), indicating repression of promoter activity. LUC enzymatic activity was quantified and showed results consistent with fluorescence imaging (Fig. S4a–d). Electrophoretic mobility shift assays (EMSAs) further validated direct DNA binding. Recombinant GST–CmBHLH93 (62 kDa) was expressed in *Escherichia coli* Rosetta (DE3) and purified (Fig. 7d). GST–CmBHLH93 bound biotin-labeled probes corresponding to the E-box regions of target promoters, producing shifted bands. This interaction was competed by a 10-fold excess of unlabeled probe, whereas mutated probes failed to compete, confirming sequence-specific binding (Fig. 7e). Together, these results demonstrate that CmBHLH93 is a nuclear transcription factor with DNA-binding activity toward E-box motifs in the promoters of *CmNCED3*, *CmAOS*, *CmPOD-1*, and *CmSOS1*, where it functions as a transcriptional repressor of these stress-responsive genes.

**Figure 7:**
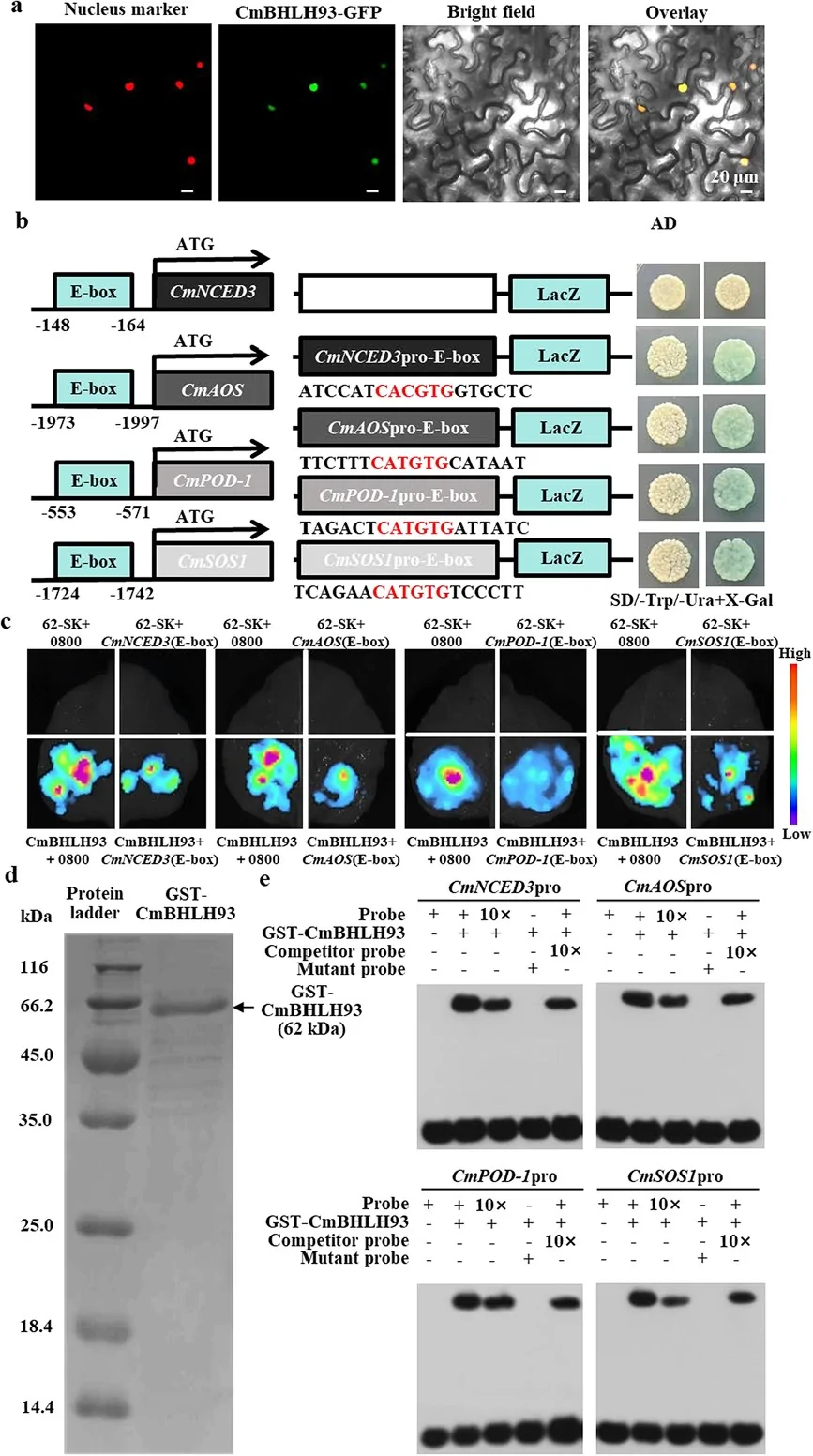
Interaction analysis of CmBHLH93 with downstream gene promoters. (a) Subcellular localization of CmBHLH93-GFP fusion protein in *N. benthamiana* epidermal cells, showing exclusive nuclear localization. The nuclear marker mCherry-NLS was co-expressed as a positive control. Scale bar = 20 μm. (b) Yeast one-hybrid (Y1H) assay showing specific binding of GAL4 activation domain (AD)–fused CmBHLH93 to triple tandem repeats of E-box elements (CANNTG) from the promoters of *CmNCED3*, *CmAOS*, *CmPOD-1*, and *CmSOS1*. Empty AD vector with corresponding promoter constructs served as negative controls. (c) Dual-luciferase reporter assay in *N. benthamiana* leaves showing transcriptional repression activity of CmBHLH93. (d) SDS-PAGE analysis of purified recombinant GST-CmBHLH93 fusion protein (expected molecular mass: 62 kDa) expressed in *E. coli* Rosetta (DE3) and affinity-purified using glutathione resin. M, protein molecular weight marker. (e) Electrophoretic mobility shift assay (EMSA) confirming sequence-specific binding of GST-CmBHLH93 to biotin-labeled E-box probes. Competition with 10× molar excess of unlabeled (cold) probe abolished the shifted complex, whereas mutated E-box probes (core sequence replaced with A residues) failed to compete, demonstrating binding specificity.

## Discussion

### CmbHLH93 negatively regulates salt–alkali tolerance in melon seedlings enhanced by nano-Fe₃O₄

The bHLH family is one of the largest transcription factor families in plants and exhibits substantial functional divergence, with members acting as either positive or negative regulators of stress responses [40]. For example, overexpression of *AtbHLH122* in *Arabidopsis* enhances salt and drought tolerance, whereas the *bhlh122* loss-of-function mutant shows increased sensitivity to salt and osmotic stress, indicating a positive regulatory role [53]. Similarly, BEAR1 positively regulates salt responses in rice, as evidenced by the salt-sensitive phenotypes of knockout lines [54]. In contrast, the *OsbHLH024* knockout mutant (A91) exhibited increased shoot biomass and antioxidant activity under salt stress, suggesting a negative regulatory function [52]. In this study, CmBHLH93 was identified as a bHLH transcription factor that acts as a negative regulator of salt–alkali tolerance in melon. Knockout of *CmBHLH93* (KOBHLH93) significantly improved growth under salt–alkali stress, while overexpression (OEBHLH93) lines were more sensitive (Fig. 2  b–e). Consistently, KOBHLH93 seedlings exhibited reduced wilting after 5 h of stress, whereas OEBHLH93 seedlings showed more severe wilting. These findings indicate that CmbHLH93 negatively regulates salt–alkali tolerance in melon seedlings.

To explore whether nano-Fe₃O₄ acts through CmBHLH93, foliar application was performed on KOBHLH93 and OEBHLH93 lines under salt–alkali stress. Nano-Fe₃O₄ treatment further enhanced growth in KOBHLH93 lines, relative to EV controls, exceeding the increases observed under water treatment alone. In contrast, OEBHLH93 lines exhibited stronger growth inhibition under nano-Fe₃O₄ treatment, compared with more moderate reductions under water treatment (Fig. 2b–e). These results suggest that CmBHLH93 modulates the magnitude of the nano-Fe₃O₄ response under salt–alkali stress. Suppression of CmBHLH93 enhances the beneficial effects of nano-Fe₃O₄, whereas its overexpression attenuates these effects. Overall, these findings establish CmBHLH93 as a negative regulator of salt–alkali tolerance in melon and implicate it as a key component of the nano-Fe₃O₄–mediated stress response pathway.

### CmBHLH93 negatively regulates melon salt–alkali tolerance by inhibiting abscisic acid and jasmonic acid biosynthesis via direct binding to the CmNCED3 and CmAOS promoters

ABA and JA are key phytohormones that regulate plant growth, development, and stress responses [55]. Under salt–alkali stress, rapid accumulation of ABA and JA activates defence pathways, including stomatal regulation and stress adaptation mechanisms [56, 57]. Transcriptomic analysis in this study identified significant enrichment of plant hormone signaling pathways, implicating CmBHLH93 in the regulation of ABA- and JA-associated responses. Consistent with this, KOBHLH93 lines exhibited elevated endogenous ABA and JA levels, whereas OEBHLH93 lines showed reduced hormone levels. Under salt–alkali stress, nano-Fe₃O₄ treatment further enhanced ABA and JA accumulation in KOBHLH93 lines, while OEBHLH93 lines maintained lower hormone levels. These findings indicate that nano-Fe₃O₄ enhances ABA and JA accumulation, at least in part, through suppression of *CmBHLH93*, consistent with its negative regulatory function.

Mechanistically, CmBHLH93 directly binds to E-box motifs in the promoters of the ABA biosynthesis gene *CmNCED3* and the JA biosynthesis gene *CmAOS*, repressing their transcription. NCED (9-*cis*-epoxycarotenoid dioxygenase) catalyse a rate-limiting step in ABA biosynthesis [58], and their expression is rapidly induced under salt stress conditions [59]. In agreement with this, *CmNCED3* expression increased in KOBHLH93 lines and decreased in OEBHLH93 lines, consistent with the observed changes in ABA levels. Among the four NCED family genes in melon, *CmNCED3* is primarily expressed in stems, female flowers, and tendrils, with relatively low expression in leaves. However, *CmNCED*3 is upregulated in melon leaves under various abiotic stresses, indicating its role in stress responses [60]. In the present study, *CmNCED3* expression showed a more pronounced increase in roots than in leaves, suggesting tissue-specific regulation under salt–alkali stress. In addition, another ABA biosynthesis gene, *CmABA1*, was significantly upregulated in the leaves of KOBHLH93 lines. This discrepancy may reflect differences in spatial and temporal dynamics of ABA biosynthesis under stress conditions. Since roots act as primary stress sensors of salinity, rapid induction of *CmNCED3* in roots likely represents an early, local defense mechanism that initiates root-to-shoot ABA signaling [61].

Allene oxide synthase (AOS) catalyzes a key step in JA biosynthesis and represents an important regulatory node in the pathway [62, 63]. In rice, *OsAOS1* and *OsAOS2* expression are induced by mechanical wounding, herbivore attack, and JA treatment [64]. Mechanical wounding, MeJA application, and H₂O₂ treatment induce *CmAOS* expression in melon. Inoculation with the powdery mildew pathogen *Podosphaera xanthii* further enhances *CmAOS* expression, indicating its function in activating JA-mediated defense against fungal infection [65]. In the JA biosynthetic pathway, Lipoxygenase (LOX) catalyzes the conversion of alpha-linolenic acid to 13-HPOT, which is subsequently converted by AOS, placing AOS immediately downstream of LOX [66]. In our study, *CmLOX2-2* expression increased in KOBHLH93 lines in leaves and *CmLOX2-1* expression increased in roots, whereas overexpression had the opposite effect. Importantly, CmBHLH93 directly binds to the promoter of *CmAOS* and represses its transcription. The coordinated changes in *LOX* and *AOS* expression likely reflect pathway-level regulation rather than direct targeting of *LOX* by CmBHLH93.

In the broader stress signaling network, bZIP and MYB transcription factors positively regulate ABA and JA pathways. These factors activate ABA biosynthetic genes, including *NCED3*, or downstream responsive genes by interacting with ABRE or G-box *cis*-elements. For instance, AtMYB96 and OsbZIP23 have been shown to directly bind to the promoters of *NCED* genes to form a positive feedback loop for ABA accumulation [67, 68]. In contrast, CmBHLH93 functions as a transcriptional repressor, highlighting a negative regulatory layer in hormone biosynthesis. This suggests that precise control of hormone homeostasis under stress involves both activation and repression mechanisms. Taken together, these findings indicate that nano-Fe₃O₄ enhances salt–alkali tolerance by relieving CmBHLH93-mediated repression of ABA and JA biosynthesis, thereby promoting hormone accumulation and downstream stress responses.

### CmBHLH93 negatively regulates melon salt tolerance by inhibiting POD enzyme activity and Na^+^ transport via direct regulation of *CmPOD-1* and *CmSOS1*

Salt–alkali stress imposes two primary physiological challenges: oxidative damage from ROS overproduction and ionic toxicity. bHLH transcription factors are known to modulate both processes under salt stress. For example, *ThbHLH1* is highly expressed, resulting in a significant increase in the activities of POD and SOD in *Tamarix hispida* [69]. In *Arabidopsis*, AtbHLH112 increases the expression of the *POD* and *SOD* genes to confer salt tolerance [53]. In rice, OsbHLH062 regulates ion transport genes such as *OsHAK21* and modulates the JA pathway, thereby enabling plants to withstand salt stress [70]. Moreover, AtbHLH122 regulates ABA-mediated expression of *AtNHX1* and *AtNHX6*, both of which function as Na^+^/H^+^ exchangers to increase salt tolerance in *Arabidopsis* [71]. In this study, GO enrichment analysis of differentially expressed genes in both knockout and overexpression lines revealed significant enrichment in transporter activity and redox activity (Fig. 4), indicating that CmBHLH93 is associated with these key defense pathways.

In terms of antioxidant defense, CmBHLH93 functions as a negative regulator. OEBHLH93 lines exhibited higher MDA levels, indicative of increased lipid peroxidation, together with reduced activities of key antioxidant enzymes, including SOD, POD, and CAT, compared with KOBHLH93 and control lines (Fig. 5). These results indicate that CmBHLH93 suppresses ROS-scavenging systems, whereas nano-Fe₃O₄ alleviates oxidative damage, at least in part, by repressing *CmBHLH93* expression. Mechanistically, CmBHLH93 directly binds to *CmPOD-1* promoter and represses its expression. POD plays a central role in H₂O₂ detoxification and contributes to cell wall reinforcement and stress defense [21]. Repression of *CmPOD-1* by CmBHLH93 therefore provides a mechanistic explanation for the reduced POD activity and increased oxidative damage observed in overexpression lines.

In parallel, CmBHLH93 negatively regulates ion homeostasis. Maintaining a low intracellular Na^+^/K^+^ ratio is essential for plant salt tolerance. KOBHLH93 lines exhibit a lower Na^+^/K^+^ ratio under salt–alkali stress, whereas OEBHLH93 lines showed impaired regulation (Fig. 6). This phenotype is consistent with transcriptional control of the Na^+^ efflux transporter CmSOS1. *CmSOS1* encodes a plasma membrane Na^+^/H^+^ antiporter that extrudes Na^+^ from the cytoplasm and is a core component of the SOS pathway [7, 9]. We show that CmBHLH93 directly binds to the *CmSOS1* promoter and represses its transcription. Reduced *CmSOS1* expression in OEBHLH93 lines is therefore consistent with increased Na^+^ accumulation and disrupted ionic homeostasis, whereas enhanced expression in knockout lines supports improved Na^+^ exclusion.

The transcriptional regulation of *SOS1* is known to involve both positive and negative regulators; for instance, AtMYB74 activates *SOS1* expression under salt stress [72]. In this context, CmBHLH93 represents a negative regulatory component. The suppression of CmBHLH93 by nano-Fe₃O₄ likely relieves this repression, thereby facilitating activation of Na^+^ efflux and restoration of ionic balance.

## Conclusion

In summary, nano-Fe₃O₄ treatment significantly suppressed the expression of *CmBHLH93*. As a negative regulator, CmBHLH93 directly binds to the promoters of key defense-related genes, including *CmNCED3* (ABA synthesis), *CmAOS* (JA synthesis), *CmPOD-1* (antioxidant defense), and *CmSOS1* (ion transport), thereby repressing their expression and reducing salt–alkali tolerance in melon seedlings. By suppressing *CmBHLH93* expression, nano-Fe₃O₄ activates downstream defense responses involving ABA/JA signaling, antioxidant systems, and ion homeostasis, ultimately enhancing salt–alkali tolerance in melon seedlings (Fig. 8).

**Figure 8:**
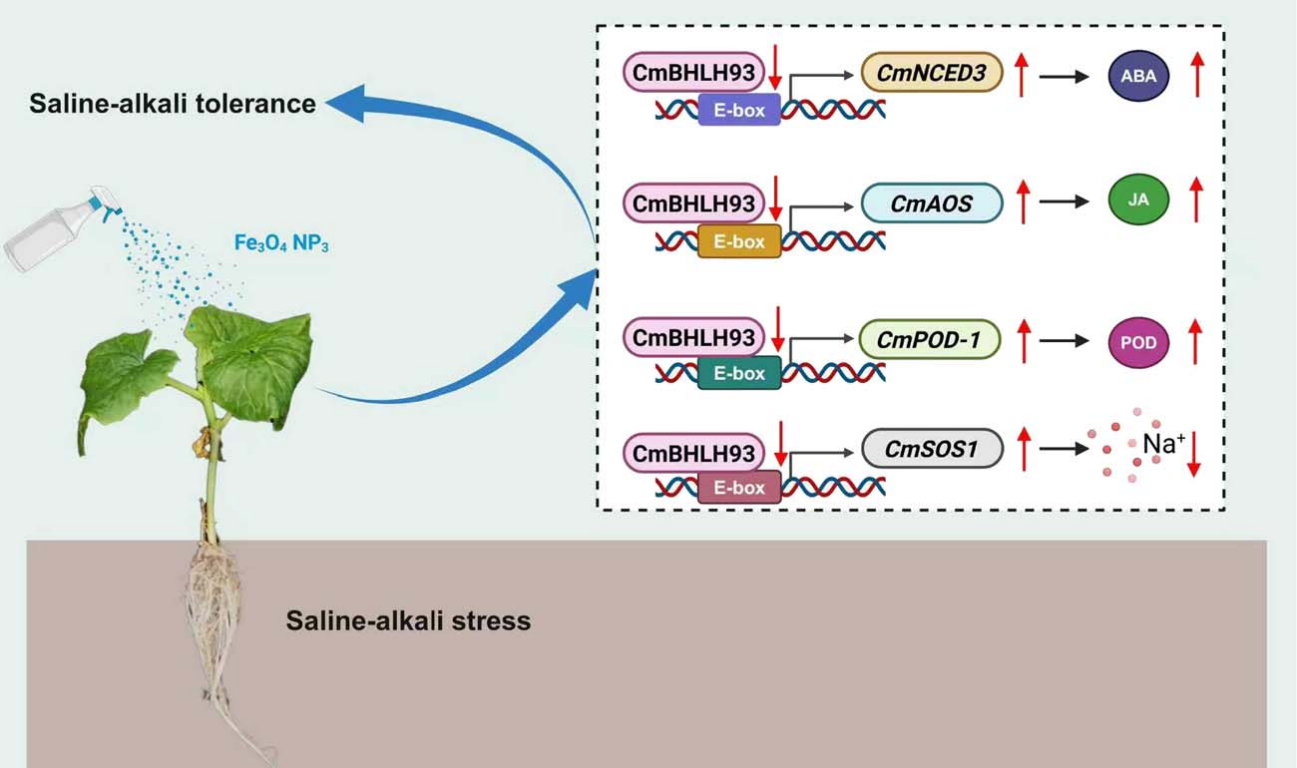
Molecular mechanism by which CmBHLH93 regulates nano-Fe_3_O_4_-mediated enhancement of salt–alkali tolerance in melon seedlings. Schematic illustration of the regulatory cascade. Under salt–alkali stress, exogenous nano-Fe₃O₄ (100 mg/L, foliar application) suppresses the expression of *CmBHLH93*, a nucleus-localized bHLH transcription factor functioning as a transcriptional repressor. Downregulation of CmBHLH93 reduces its binding to E-box motifs (CANNTG) in the promoters of four key defense genes: *CmNCED3* (ABA biosynthesis), *CmAOS* (JA biosynthesis), *CmPOD-1* (antioxidant defense), and *CmSOS1* (Na^+^ extrusion). Relief of CmBHLH93-mediated repression leads to the upregulation of ABA/JA signaling pathways, enhanced ROS scavenging through increased POD activity, and improved SOS1-mediated Na^+^ efflux, collectively enhancing salt–alkali tolerance in melon seedlings.

## Materials and methods

### Quantification of the CmBHLH93 protein abundance

The melon (*Cucumis melo*) cultivar used in this study was ‘XSM’, obtained from Jintudi Seed Industry Co., Ltd., Changji, Xinjiang. The seed treatment procedure was as follows: seeds were first disinfected in sterile water at 55°C for 15 min, followed by soaking in sterile water at 25°C for 6 h. Seeds were transferred to 10 cm Petri dishes lined with three layers of moist filter paper and germinated at 28°C. Germinated seeds were then sown onto sponge blocks soaked in half-strength Hoagland’s nutrient solution. At the two-leaf stage, seedlings were transplanted into hydroponic cups (90 mm top diameter, 57 mm bottom diameter, 135 mm height) containing nutrient solution without added salt or alkali as the control. The nutrient solution consisted of half-strength modified Hoagland’s solution containing 1 mM MgSO_4_•7H_2_O, 4 mM CaCl_2_, 60 mM KNO_3_, 0.5 mM Ca (H_2_PO_4_)_2_•H_2_O, and 150 mg/L Coolaber trace element solids (DZPM0059-500G) [73]. The pH was adjusted to 6.8 using KOH. After 24 h of hydroponic culture, leaves were sprayed with nano-Fe_3_O_4_ for 24 h, followed by 5 h of 200 mM salt–alkali stress treatment (final salt concentration of 200 mM; NaCl: Na₂SO₄: NaHCO₃: Na₂CO₃ = 1: 9: 9: 1, pH = 8.9). Root tissues were harvested for proteomic profiling. The proteomic analysis was outsourced to Shanghai Majorbio Bio-Pharm Technology Co., Ltd. Label-free quantitative proteomics was performed on an LC-MS/MS platform. Briefly, total proteins were extracted from root tissues and subjected to reduction, alkylation, trypsin digestion, and peptide desalting. The resulting peptides were separated using an EASY-nLC 1200 nano-flow liquid chromatography system, followed by data-independent acquisition (DIA) on a Thermo Fisher Orbitrap high-resolution mass spectrometer. Raw data were processed with Spectronaut software for database searching, protein identification, quality control, and relative quantification. Proteomic data are provided in Table S1.

### Melon seedling culture and generation of root-transformed plants

The melon cultivar, seed disinfection, germination, and hydroponic seedling culture conditions were the same as described in the Quantification of the CmBHLH93 Protein Abundance section. When the cotyledons were fully expanded, *Agrobacterium*-mediated genetic transformation (strain GV3101) was used to generate root-transformed lines with *CmBHLH93* knockout (KOBHLH93) and overexpression (OEBHLH93). Plants transformed with an EV were used as controls. Successful overexpression and gene editing were confirmed by qRT-PCR (for OEBHLH93) and Hi-TOM analysis (for KOBHLH93), respectively. At the two-leaf stage, seedlings were transplanted into hydroponic cups containing the same half-strength modified Hoagland’s nutrient solution as in the Quantification of the CmBHLH93 Protein Abundance section. After 24 h of hydroponic culture, leaves were sprayed with nano-Fe₃O₄ for 24 h, followed by a 5-h salt–alkali stress treatment (200 mM; NaCl:Na₂SO₄:NaHCO₃:Na₂CO₃ = 1:9:9:1, pH = 8.9), as detailed in the Quantification of the CmBHLH93 Protein  Abundance section. After treatment, samples were collected to measure growth parameters (fresh and dry weight of shoots and roots) and physiological indicators (malondialdehyde content, hormones, and antioxidant enzyme activities). For physiological assays, all treatments included three biological replicates, each consisting of six independent seedlings.

### Measurement of physiological indicators

After leaves were sprayed with nano-Fe_3_O_4_ for 24 h, melon seedlings were subjected to 5 h of salt–alkali stress, and samples were collected for the measurement of related physiological indicators.

#### Malondialdehyde content

Leaf samples (0.2 g) were collected, immediately frozen in liquid nitrogen, and thoroughly ground. Then, 5 ml of precooled 10% trichloroacetic acid (TCA) solution was added for extraction, and the mixture was centrifuged to obtain the supernatant. The supernatant was mixed with 0.6% thiobarbituric acid (TBA) solution, and a color reaction was performed under specific conditions. Finally, the absorbance of the reaction solution was measured at 532 and 600 nm using a spectrophotometer, and the MDA accumulation in the sample was calculated based on the absorbance values.

#### Measurement of hormone content

ABA and JA were quantified using an ultra-high performance liquid chromatography–tandem mass spectrometry system (Agilent 1290 UHPLC-6460 Triple Quad). Approximately 0.1 g of plant tissue was frozen in liquid nitrogen and combined with 1 ml of precooled aqueous formic acid supplemented with isotope-labeled internal standards. The sample was ground and centrifuged at 12 000 × *g* for 15 min at 4°C. The supernatant was then purified using a C18 solid-phase extraction column, concentrated under a nitrogen stream, and reconstituted in 100 μl of methanol. Chromatographic separation was performed on a ZORBAX RRHD Eclipse Plus C18 column (2.1 × 100 mm, 1.8 μm) at 35°C with a flow rate of 0.25 ml·min^−1^. The mobile phases were 0.1% aqueous formic acid (A) and acetonitrile (B). The gradient elution program was as follows: 0–2 min, 10% B; 2–6 min, 10% → 40% B; 6–8 min, 40% → 95% B; 8–10 min, 95% B; 10–10.1 min, 95% → 10% B; 10.1–12 min, 10% B. Mass spectrometric detection was conducted using an electrospray ionization source with positive/negative ion switching mode and multiple reaction monitoring (MRM). The ion source parameters were: drying gas temperature 300°C, flow rate 8 L·min^−1^, nebulizer pressure 35 psi, and capillary voltage ±3.5 kV. Each hormone was qualitatively identified based on two characteristic ion pairs and quantitatively determined using the internal standard method.

#### Measurement of antioxidant enzyme activities

All measurements were performed using biochemical assay kits provided by Jiangsu Aidisheng Biotechnology Co., Ltd. The specific kit models were ADS-W-YH002 for MDA, ADS-W-KY011 for superoxide dismutase (SOD), ADS-W-KY003 for peroxidase (POD), and ADS-W-KY002 for catalase (CAT).

#### Measurement of sodium and potassium content

Zero-point two grams of fully expanded true leaves with veins removed was collected. Samples were first heated at 105°C for 30 min to inactivate enzymes, then dried in an oven at 80°C until constant weight. Dried samples were placed into digestion vessels and treated with 10 ml of a nitric acid–hydrogen peroxide mixture (V:V = 9:1) for microwave digestion. The digested solution was then diluted to 25 ml using ultrapure water. Sodium and potassium contents were measured using a Shimadzu AA-6880 atomic absorption spectrophotometer at wavelengths of 589 nm (Na^+^) and 766 nm (K^+^). Ion concentrations (mg·g^−1^ dry weight) were calculated based on standard curves, and the sodium-to-potassium ratio (Na^+^/K^+^) was subsequently determined.

#### Transcriptome analysis

Melon seedlings were sprayed with nano-Fe₃O₄ for 24 h and then exposed to 5 h of salt–alkali stress. The second true leaf from the apex was harvested immediately, rapidly frozen in liquid nitrogen, and stored at −80°C. Each treatment comprised three biological replicates, each containing over 10 seedlings. Total RNA was extracted using the Trizol method. Subsequently, paired-end (PE150) sequencing libraries were constructed and sequenced on the Illumina NovaSeq 6000 platform. Raw sequencing data were quality-filtered using Trimmomatic to obtain high-quality reads. Clean reads were aligned to the melon reference genome (DHL92 v4.0) using Hisat2, and gene expression levels were estimated with featureCounts. DEGs were identified using the DESeq2 method. Transcriptome data have been uploaded to NCBI (PRJNA1399975).

### qRT-PCR analysis

Following the transcriptome experimental procedure, total RNA was extracted using the RNAsimple Total RNA Extraction Kit [Tiangen Biotech (Beijing) Co., Ltd.] and used as a template for cDNA synthesis. In the quantitative PCR (qPCR) experiments, *CmActin* was used as the endogenous reference gene. The transcription levels of target genes were detected using SYBR Green qPCR premix (Grisi Bio) on an ABI QuantStudio 6 Pro real-time PCR system. Each reaction system had a total volume of 10 μl, comprising 5 μl SYBR Green Mix, 0.4 μl forward primer, 0.4 μl reverse primer, 1 μl cDNA template, and 0.2 μl ROX reference dye. The PCR amplification program was set as follows: initial denaturation at 95°C for 30 s, followed by 40 cycles of 95°C denaturation for 5 s and 60°C annealing/extension for 30 s. The relative expression levels of target genes were calculated using the 2^−ΔΔCt^ method. Sequences of primers for relevant genes are listed in Table S2.

### Subcellular localization

The *CmBHLH93* coding sequence without stop codon was ligated into the 1305.4-GFP vector (EcoRI and SalI sites) to generate the CmBHLH93-GFP recombinant plasmid. Competent *Agrobacterium tumefaciens* GV3101 cells were transformed with the recombinant plasmid. The transformed culture was combined with *Agrobacterium* containing the nuclear marker mCherry and infiltrated into *N. benthamiana* leaves for transient expression. Infiltrated plants were incubated under standard greenhouse conditions for 2–3 days, and fluorescence signals were examined using a laser scanning confocal microscope. Primer sequences for vector construction are provided in Table S3.

### Yeast one-hybrid assay

In the yeast one-hybrid (Y1H) assay, the pB42AD vector was first linearized using EcoRI and XhoI restriction enzymes, and the *CmBHLH93* coding sequence (CDS) fragment was ligated into the vector at the corresponding restriction sites. BHLH-binding sequences (E-box) from the promoters of *CmNCED3*, *CmAOS*, *CmPOD-1*, and *CmSOS1* were synthesized by Beijing Tsingke Biotechnology Co., Ltd, and three tandem repeats of each sequence were inserted into the pLacZi vector. The constructed pB42AD-CmBHLH93 plasmid and pLacZi-E-box were co-transformed into EGY48 yeast cells. Yeast co-transformed with pB42AD empty vector and pLacZi-E-box served as the negative control. Single clones were cultured on SD/-Trp/-Ura solid medium and then inoculated into SD/-Trp/-Ura liquid medium, followed by overnight incubation at 28°C with shaking at 200 rpm. Following incubation, the yeast suspension was adjusted to an OD_600_ of 0.1. Subsequently, 10 μl of the suspension was spotted onto SD/-Trp/-Ura/Gal/Raf/X-Gal solid medium. Plates were incubated at 30°C for 3–5 days, and the interaction was assessed based on colony color development. Sequences of primers for vector construction are listed in Table S3.

### LUC

The LUC assay was performed following previously reported methods [73]. Primers were designed to amplify the *CmBHLH93* CDS, and the amplified fragment was inserted into the BamHI and XhoI sites of the pGreenII62-SK vector. BHLH-binding sequences (E-box) from the promoters of *CmNCED3*, *CmAOS*, *CmPOD-1*, and *CmSOS1* were synthesized by Beijing Tsingke Biotechnology Co., Ltd, and three tandem repeats were inserted into the pGreenII0800-LUC vector via KpnI and SmaI sites. The constructed pGreenII62-SK-CmBHLH93 and pGreenII0800-LUC-E-box were co-transformed into *A. tumefaciens* GV3101 and subsequently infiltrated into *N. benthamiana* leaves. Transformed *N. benthamiana* plants were grown under dark conditions for 2–5 days, after which the abaxial leaf surface was sprayed with D-Luciferin potassium salt (Biyuntian, Shanghai). Fluorescence was detected using a NightSHADE LB985 imaging system (Berthold, Germany). Dual-LUC reporter gene test kits from Beyotime (Shanghai, China) were used to determine the LUC/REN ratio. Sequences of primers for vector construction are listed in Table S3.

### Electrophoretic mobility shift assay

The *CmBHLH93* CDS was first cloned into the pGEX-4 T-2 plasmid. GST-CmBHLH93 recombinant protein was expressed in BL21 *E. coli* Rosetta (DE3) induced with 0.2 mM IPTG at 16°C and purified using GST-Sefinose™ resin. Biotin-labeled probes containing BHLH-binding sequences (E-box) from the promoters of *CmNCED3*, *CmAOS*, *CmPOD-1*, and *CmSOS1* were synthesized by Beijing Tsingke Biotechnology Co., Ltd, and annealed to generate biotin-labeled double-stranded DNA. Binding reactions were performed using purified GST-CmBHLH93 protein and biotin-labeled probes, with or without competition from a 10-fold molar excess of unlabeled (cold) probes. Reactions were incubated at room temperature and then resolved on a nondenaturing polyacrylamide gel in 0.5× TBE buffer. After electrophoresis, DNA–protein complexes were transferred to a nylon membrane and UV-crosslinked. Signals were detected using a chemiluminescent EMSA detection kit according to the manufacturer’s instructions. Sequences of primers for vector construction are listed in Table S3.

### Data statistics and analysis

All data in this study were subjected to two-way analysis of variance (two-way ANOVA) with a significance level set at *P* < 0.05. When interaction effects or main effects reached significance, multiple comparisons were further performed using Duncan’s new multiple range test (DMRT). Data analysis and visualization were conducted in the RStudio 4.0.3 environment, with all referenced gene annotations provided in Table S4.

## Supplementary Material

Web_Material_uhag229

## Data Availability

The authors declare that all the data necessary to support the study’s conclusions are included in the paper and the supplemental materials or can be obtained upon request from the corresponding author.
